# INTEREST GROUP SESSION—ECONOMICS OF AGING: EVOLVING CARE SYSTEMS FOR OLDER ADULTS: UTILIZATION IMPACTS OF MEDICARE AND MEDICAID PAYMENT TRENDS

**DOI:** 10.1093/geroni/igz038.2015

**Published:** 2019-11-08

**Authors:** Christine E Bishop

**Affiliations:** Brandeis University, Waltham, Massachusetts, United States

## Abstract

With the promise of better care coordination, better member outcomes, and lower costs, Medicare and state Medicaid programs are implementing population-based payment systems for older adults. Medicare Advantage (MA) plans are responsible for Medicare services for their members, Medicaid managed long-term services and supports (MLTSS) programs cover a broad span of Medicaid benefits, and some state initiatives enroll beneficiaries dually eligible for both Medicaid and Medicare and integrate benefits from the two programs. Simultaneously, Medicaid programs are attempting to shift LTSS utilization away from nursing homes and toward home and community based services (HCBS). The presentations for this symposium address aspects of this changing landscape using Medicare and Medicaid claims and other data and causal econometric models. The first paper considers the effect of MA utilization on SNF staffing, quality, and financial health. The second paper compares medical care utilization outcomes, specifically risk of hospitalization, for Medicaid nursing home residents to outcomes for similar Medicaid members receiving HCBS. The third paper presents an MLTSS initiative in one state in the context of national developments and considers the challenges of evaluating its impact. The fourth paper compares hospitalization rates for MLTSS populations to rates for dually eligible-beneficiaries not enrolled in MLTSS. The discussion will bring findings together to assess early gains and costs as these systems of care evolve.

